# Reduced Dehydroepiandrosterone-Sulfate Levels in the Mid-Luteal Subphase of the Menstrual Cycle: Implications to Women’s Health Research

**DOI:** 10.3390/metabo12100941

**Published:** 2022-10-04

**Authors:** Ajna Hamidovic, Fatimata Soumare, Aamina Naveed, John Davis, Jiehuan Sun, Nhan Dang

**Affiliations:** 1 Department of Pharmacy, University of Illinois at Chicago, Chicago, IL 60612, USA; 2 Department of Psychiatry, University of Illinois at Chicago, Chicago, IL 60612, USA; 3 Department of Public Health, University of Illinois at Chicago, Chicago, IL 60612, USA

**Keywords:** dehydroepiandrosterone-sulfate, DHEA-sulfate, DHEA, steroid sulfotransferase, SULT, steryl-sulfatase, STS, menstrual cycle, follicular, luteal

## Abstract

The regulation of DHEA-sulfate by steroid sulfotransferase (SULT) and steryl-sulfatase (STS) enzymes is a vital process for the downstream formation of many steroid hormones. DHEA-sulfate is the most abundant steroid hormone in the human body; thus, DHEA-sulfate and its hydrolyzed form, DHEA, continue to be evaluated in numerous studies, given their importance to human health. Yet, a basic question of relevance to the reproductive-age female population—whether the two steroid hormones vary across the menstrual cycle—has not been addressed. We applied a validated, multi-step protocol, involving realignment and imputation of study data to early follicular, mid-late follicular, periovulatory, and early, mid-, and late luteal subphases of the menstrual cycle, and analyzed DHEA-sulfate and DHEA serum concentrations using ultraperformance liquid chromatography tandem mass spectrometry. DHEA-sulfate levels started to decrease in the early luteal, significantly dropped in the mid-luteal, and returned to basal levels by the late luteal subphase. DHEA, however, did not vary across the menstrual cycle. The present study deep-mapped trajectories of DHEA and DHEA-sulfate across the entire menstrual cycle, demonstrating a significant decrease in DHEA-sulfate in the mid-luteal subphase. These findings are relevant to the active area of research examining associations between DHEA-sulfate levels and various disease states.

## 1. Introduction

**Figure 1:**
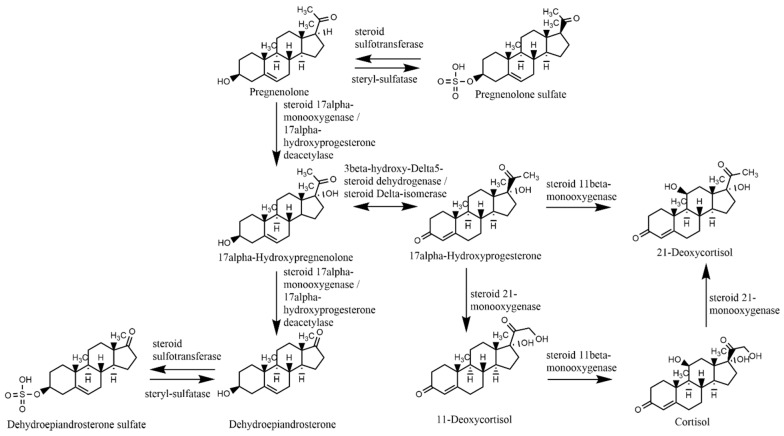
Biochemical pathways for the synthesis of dehydroepiandrosterone and dehydroepiandrosterone-sulfate.

Dehydroepiandrosterone-sulfate (DHEA-sulfate) is the most abundant hormone in the peripheral circulation [1,2,3]. It is synthesized from dehydroepiandrosterone (DHEA) by steroid sulfotransferase (SULT) and hydrolyzed back to DHEA by steryl-sulfatase (STS) (Figure 1). DHEA-sulfate and DHEA rise with adrenarche and approach the nadir around the age of 70 [4,5]. Referred to collectively as DHEAS, DHEA and DHEA-sulfate protect against a number of diseases, including affective and psychotic disorders, substance use disorder, cancer, diabetes, and obesity [6]. The effects of DHEA on breast cancer development are complex, and can be tumor promoting as well as tumor suppressing, depending on the system the hormone interacts with (reviewed in Chatterton [7]).

Despite the importance of DHEAS in health and disease, it is not clear how they fluctuate themselves as well as in relation to one another across the six subphases of the menstrual cycle. The two menstrual cycle phases—follicular and luteal—are separated by ovulation. The shifts in circulating sex hormones further divide the menstrual cycle into six subphases: (1) early follicular, (2) mid-follicular, (3) periovulatory, (4) early luteal, (5) mid-luteal, and (6) late luteal [8,9].

Deep mapping of DHEAS fluctuations across the menstrual cycle is relevant given the steroids’ neuro-stimulatory and anti-glucocorticoid effects [10], and the resulting behavioral changes [6]. For example, antidepressant actions of DHEA-sulfate are attributed to the δ-subunit containing GABA_A_ receptors in the nucleus accumbens and ventral tegmental area [11], with the perfusion of DHEA-sulfate into those regions improving swim test performance of Flinders Sensitive Line rats. Moreover, sigma-1 receptor stimulation by DHEA increases neurogenesis in the hippocampal dentate gyrus of mice and ameliorates depressive-like behaviors [12].

Antidepressant effects shown in animal models translate to humans, with positive effects of DHEA therapy on mood [13,14] and negative symptoms in schizophrenia patients [15]. With some exceptions [6,16], higher levels of DHEAS are associated with lower depression scores [17,18,19,20,21]. Higher DHEAS levels are also found in healthy controls relative to major depressive disorder (MDD) patients [19]. The relevance of DHEA-sulfate extends to additional affective disorders. For example, administration of Lupron, which suppresses ovarian secretion of the sex hormones estradiol and progesterone, followed by an add-back treatment of progesterone, results in lower levels of DHEA-sulfate in premenstrual dysphoric disorder (PMDD) patients vs. healthy controls, implicating the metabolism of DHEA-sulfate in the etiology of PMDD [22]. In summary, past findings demonstrate that lower levels of DHEAS are generally associated with pathophysiology and worse outcomes.

The present study improves the methodology of clinical neuroactive steroid research in the context of the menstrual cycle. Circulating neuroactive steroid changes in reproductive-age women have been evaluated for a limited number of menstrual cycle subphases, which may have been mistimed due to reliance on post hoc progesterone values for mid-luteal subphase staging. The most accurate method for staging the mid-luteal and other subphases is by implementing a multi-step protocol, which involves an analysis of serum luteinizing hormone (LH) levels to correctly realign menstrual cycle subphases [8,23]. In the first step of the protocol, eight clinic visits are scheduled at key estimated menstrual cycle events based on the previous menstrual cycle duration. The eight visits are: (1) early follicular, (2) mid-follicular, (3–5) three visits during the periovulatory phase, (6) early luteal, (7) mid-luteal, and (8) late luteal. In the next step, serum LH levels from these visits are used to realign study visits, as some were inherently mistimed due to a within-woman variability in ovulation timing and menstrual cycle duration. Upon data realignment, which ensures that all women are reclassified to the same subphase, the resulting missing data is imputed [8]. This methodology is implemented to study various trajectories across the entire menstrual cycle in lieu of the daily specimen collection and analyses, which are impractical and costly.

Although the results to date show that DHEAS are implicated in the development of several disorders in women, the translation of these findings to reproductive-age patients in the context of symptom exacerbation across the mensural cycle has been scarce. As a basic step toward advancing this field of research, we evaluated the levels of DHEA, DHEA-sulfate, and their ratios in premenopausal women across all the subphases of the menstrual cycle to demonstrate the importance of subphase considerations when contrasting the levels of DHEAS in diseased vs. healthy populations in future studies. Moreover, we deemed the present analysis critical for advancing our understanding of basic physiology, as it maps the activities of SULT and STS across the entire menstrual cycle. 

## 2. Materials and Methods

### 2.1. Study Design

Premenstrual Hormonal and Affective State Evaluation (PHASE) is a single-cohort longitudinal design study with a nested human laboratory between subject human psychosocial stress experiment, designed to increase our understanding of menstrual cycle physiology. PHASE is a registered clinicaltrials.gov study (NCT03862469). The study is described in detail in Hamidovic et al. [24]. In that publication, we have described an association between neuroticism and premenstrual affective/psychological symptomatology in a larger sample of PHASE participants. The present analysis includes a subset of the study population with hormone level data. Hence, the study variables are different, though the participant sample is partially the same.

PHASE enrolls women with regular menstrual cycles to chart their symptoms using the Daily Record of Severity of Problems (DRSP) [25], menstruation timing, and urinary LH test results during three menstrual cycles. In the third menstrual cycle, in addition to DRSP collection and urinary LH testing, study participants complete: (1) blood and salivary sample collection visits at 8 different times of the menstrual cycle and (2) psychosocial stress testing in the late luteal phase. PHASE is a registered clinicaltrials.gov study (NCT03862469).

### 2.2. Study Sample

Women between the ages of 18 and 35 with regular menstrual cycles lasting 21 to 35 days were recruited from the general population using flyers, word-of-mouth referrals, and electronic media (Facebook, Instagram, and Craigslist). Study participants first completed an online survey, following which they were scheduled to complete an in-person screening. Before any collection of data, they signed a consent form, approved by the University of Illinois Human Research Protection Office (2018-1533). Study exclusion criteria were: (a) lifetime psychiatric disorder, except anxiety and depression (based on the Structured Clinical Interview for DSM Disorders (SCID)), (b) current (i.e., within the past 12 months) DSM-5 Major Depressive Disorder or an anxiety disorder (based on SCID), (c) positive urine drug screen test, (d) breath alcohol concentration >0.00%, (e) Alcohol Use Disorders Identification Test (AUDIT) score >7, (e) self-reported smoker or carbon monoxide concentration ≥ 6 ppm, (f) irregular menstrual cycle, (g) current pregnancy (urine test-verified), lactation, or a plan to become pregnant, (h) moderate or high suicide risk, (i) Shipley IQ (vocabulary standard score) < 80, (j) prescription medications, and (k) hormonal contraception.

In the study, race and ethnicity were reported, as mandated by the US National Institutes of Health (NIH), and consistent with the Inclusion of Women, Minorities, and Children policy. Individuals were categorized as American Indian or Alaska Native, Asian, Black or African American, Hispanic or Latino, Native Hawaiian or Other Pacific Islander, or White, based on the NIH Policy on Reporting Race and Ethnicity Data.

### 2.3. Study Procedures

Study participants conducted urinary self-testing of the luteinizing hormone (LH) for 3 menstrual cycles using Easy@Home urine tests. In order to read the results, study participants took a photo of the strip and uploaded it to the phone app, which read the test result and determined the LH surge as “low”, “high” or “peak”. In the present study, LH surge was implemented to guide the scheduling of study visits in the third menstrual cycle (last paragraph in this section) and not for fertility purposes.

A screenshot of the LH results was uploaded daily into REDCap by study participants. Adherence to the daily urine LH testing procedure is critical, as capturing of the short-lived peak LH surge is based on the daily reading of LH levels [26]. In the present study, adherence to LH peak testing was assessed in real time. To ensure compliance with ovulation testing, the study coordinator completed a checklist daily to monitor participant responses. In the event a participant missed a timepoint, she was contacted and asked to complete the procedure in a timely manner.

In the third menstrual cycle, study participants were scheduled to come to the clinic for 8 blood draws/saliva collection visits. Scheduling of the visits was based on the published protocol of the BioCycle study—a prospective cohort study evaluating associations between oxidative stress levels and endogenous reproductive hormone levels as well as antioxidants across the menstrual cycle in premenopausal women [8]. In the present study, the participants were given a schedule based on the average duration of their first two menstrual cycles (Supplementary Table S1). For example, a woman with a 28-day average from the previous two menstrual cycles was originally scheduled to come on days 2, 7, 12, 13, 14, 18, 22, and 27. These visits corresponded to (1) early follicular, (2) mid follicular, (3–5) three visits during the periovulatory phase, (6) early luteal, (7) mid-luteal and (8) late luteal subphases of the menstrual cycle. If a participant demonstrated a urinary LH peak prior to visit 3, she came in on the same morning and the subsequent two visits the following two days. The remaining follicular and luteal phase visits remained the same. If the urinary LH peak was detected around any other visits (or not detected), the schedule, as shown in Supplementary Table S1, remained the same.

### 2.4. Sample Collection and Analysis

DHEA and DHEA-sulfate. Analytes were dissolved in either methanol or acetonitrile to obtain a stock solution of 1 mg/mL. They were then diluted 200 ng/mL, 100 ng/mL, 50 ng/mL, 25 ng/mL, 10 ng/mL, 5 ng/mL, 1 ng/mL, 0.5 ng/mL, 0.1 ng/mL and 0.05 ng/mL using 50% methanol in water as the spiking standards to prepare the standard curve. Internal standards were diluted to 1 µg/mL as the working solution for standard curve/sample preparation. Calibrators used to construct the standard curve for positive mode analysis were prepared by taking 100 µL from each point of the standard spiking solution and drying it under a stream of nitrogen. Dried residue was dissolved in 50 mM sodium bicarbonate (50 µL, pH 10), derivatized by adding dansyl chloride (50 µL, 1 mg/mL in acetone) and incubating at 60 °C for 15 min. Internal standard was derivatized following the same procedure and used for spiking each point of the curve. Analyses were conducted using an AB SCIEX 5500 QTRAP mass spectrometer coupled with Agilent 1290 UPLC system. All samples were eluted from an Agilent Poroshell 120 EC-C18 2.7 µm column (2.1 × 100 mm) with a flow rate of 200 µL/min. The column compartment was kept at t 50 °C. The gradient was started with 95% mobile phase A (0.1% formic acid in H_2_O) for 2 min and followed by a linear gradient increase in mobile phase B (0.1% formic acid in MeOH), from 5% to 80% for 2 min and 80% to 90% over 2 min, and kept at 90% of B for 7 min, and then re-equilibrated back to the initial condition (95% A) for 3 min, resulting in a total separation time of 16 min. Mass spectrometry experiments were performed via MRM scan using electrospray ionization (ESI) in positive ion mode with an ESI spray voltage of 4.5 kV and source temperature of 500 °C.

Luteinizing Hormone. Blood specimens were allowed to clot completely at room temperature. Serum was separated from cells within 2 h of collection, and 1 mL of the serum was then transferred to an ARUP Standard Transport Tube. The method used to assay luteinizing hormone was quantitative electrochemiluminescent immunoassay. During the first incubation, 20 μL of specimen was used to form a sandwich complex between biotinylated monoclonal LH-specific antibody and monoclonal LH-specific antibody labeled with a ruthenium. During the second incubation, streptavidin labeled microparticles were added and the resulting complex bonded to the solid phase via biotin-streptavidin interaction. The reaction mixture was then aspirated into the measuring cell where the microparticles were magnetically captured onto the surface of the electrode. Unbound substances were then removed with ProCell. A voltage was applied to the electrode to induce chemiluminescent emission measured by a photomultiplier.

### 2.5. Data Realignment and Imputation

The purpose of realigning the data was to ensure that all women were reclassified to the same subphase. Since the study visits occurred at predetermined timepoints, once serum LH surge was determined post hoc, the visits were realigned, and the data were standardized according to the algorithm in Supplementary Table S2. LH surge was considered to have occurred when serum LH value reached 14.0 IU/L or greater (ARUP Laboratories). When LH peak levels were below 14.0 IU/L (but ovulation was detected; i.e., luteal progesterone levels > 5 ng/mL), visits were individually realigned as described in Mumford et al. [8].

Following realignment, the multiple imputation methodology was implemented as some of the visits were set to missing due to the realignment of study visits. Imputation of values was completed to create 10 datasets using the multivariate imputation by chained equations (MICE) package for R [27]. Data were conditioned on age, race, body mass index, and adjacent hormone measurements [8].

### 2.6. Data Analysis

Following the realignment step, we log-transformed DHEA and DHEA-sulfate values. Following imputation, we conducted pairwise comparisons of all the timepoints for both the realigned and imputed data, and we used False Discovery Rate (FDR) to correct for multiple comparisons. The significance level was set at 0.05. All analyses were completed using the R software [28].

## 3. Results

### 3.1. Realignment and Imputation Results and Study Participants

The analysis was completed on 19 participants (from the initial 21). One participant’s cycle lasted > 40 days and was considered anovulatory. Another participant was excluded because of possible contraceptive use suggested by progesterone values > 4 ng/mL on all six study visits.

Of the 19 participants, 14 had detectable mid-cycle (periovulatory) LH levels greater than 14.0 IU/L. For those participants, their highest LH was 28.17 ± 12.97 (mean ± SD). The LH peaks were captured at one of the three periovulatory phase visits (visits 3, 4, or 5) for 11 individuals. Of the remaining participants, two participants’ LH peak was captured on visit 6, and one participant’s LH peak was captured on visit 7. The neuroactive steroid data for these participants was realigned per the Biocycle study protocol (Supplementary Table S2). The remaining five study participants did ovulate (based on their luteal phase progesterone values); however, their LH peak was missed (i.e., undetectable on any of the blood draw visits). For these individuals, we realigned study visits on an individual basis, as specified in Mumford et al. (2011) Figure 2.

### 3.2. DHEA, DHEA-Sulfate, and DHEA-Sulfate: DHEA Ratio Concentrations across the Menstrual Cycle

**Figure 2:**
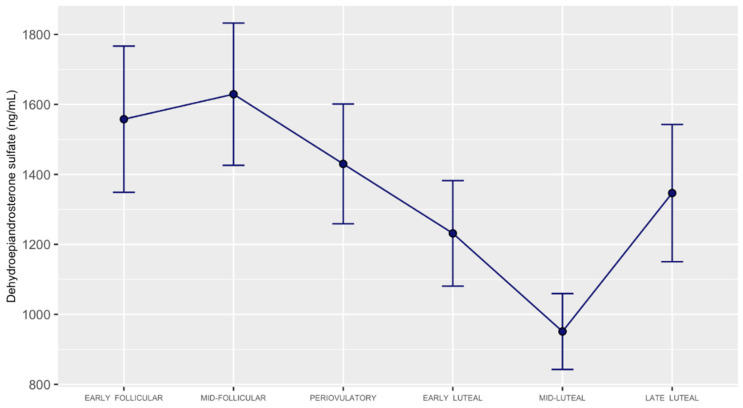
Dehydroepiandrosterone-sulfate level trajectory across the menstrual cycle in the realigned dataset. Variance is shown as standard error of the mean (SEM).

**Figure 3:**
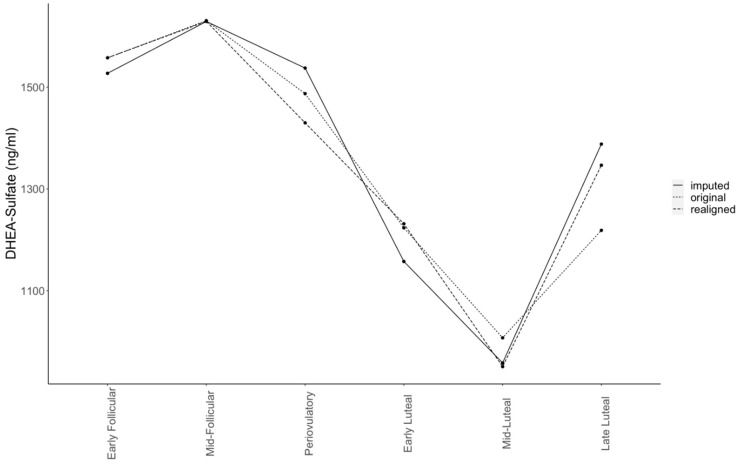
Circulating level changes for dehydroepiandrosterone-sulfate in the original, realigned, and imputed datasets. Significant differences in the realigned and imputed datasets were detected between early follicular–mid-luteal, mid-follicular–mid luteal, peri-ovulatory–mid-luteal, and mid-luteal–late-luteal subphases.

For DHEA-sulfate, we identified lower levels in mid-luteal vs. (1) early follicular, (2) mid-follicular, (3) periovulatory, and (4) late-luteal subphases (Table 1, and Figure 2 and Figure 3). We observed additional differences (only in the realigned dataset) with lower DHEA-sulfate levels for early luteal vs. (1) early follicular, (2) mid-follicular, and (3) periovulatory subphases. Results of FDR-corrected pairwise comparisons across all the timepoints indicated no significant differences in DHEA (Supplementary Table S3 and Figure S1). DHEA-sulfate: DHEA ratio was significant in the realigned dataset for the mid-follicular—mid-luteal pairwise comparison and marginally significant for the mid-luteal vs. early follicular, mid-follicular, and periovulatory subphases (Supplementary Table S4 and Figure S2).

## 4. Discussion

In the present analysis, we found that the mid-luteal subphase of the menstrual cycle is marked by the lowest levels of DHEA-sulfate. We did not find that DHEA varies significantly across the menstrual cycle. The ratio of DHEA-sulfate to DHEA was significantly decreased in the mid-luteal subphase in the realigned dataset and marginally decreased in the imputed dataset, which included covariates.

Regulation of DHEA-sulfate by SULT and STS enzymes is a vital process for the downstream formation of many steroid hormones. For example, estrogens can be formed from DHEA-sulfate (reviewed in Rizner [29]), and a perturbation of the delicate balance regulating DHEA-sulfate can lead to increased estrogen levels along with the development of several associated gynecological disease states. In a study by Colette et al. [30], there was no significant increase in STS gene and mRNA expression observed in the endometrium in the luteal relative to the follicular phase in healthy participants. At the protein level, however, there was a significant downregulation observed in the late luteal subphase (i.e., late secretory subphase) in a study by Dassen et al. [31]. Moreover, of the three enzymes which form DHEA-sulfate—SULT2A1, SULT2B1a, and SULT2B1b, with relatively similar Km [32,33]—the levels of SULT2B1b transcript were found to be increased in the endometrial stromal cells upon progesterone treatment [34]. This effect of progesterone in the secretory phase is similar to its effect on the related sulfotransferase SULT1E1, which forms estradiol-sulfate, thereby antagonizing the effect of estradiol in the endometrium [31,35]. This physiological milieu, at least with respect to SULT2B1b, favors an intracellular increase in DHEA-sulfate in the mid-luteal subphase when progesterone levels peak. Moreover, and further contributing to this increase in intracellular DHEA-sulfate content is the finding that, under the condition of estradiol priming (as in the luteal phase), progesterone increases sulfate uptake into endometrial epithelial cells [36]. This phenomenon is due to progesterone’s potent effect on the accumulation of intracellular cyclic AMP, which can be observed over either the short or long term, is independent of transcriptional or translational events, and is, at a minimum, a four-fold increase [37,38]. However, as more intracellular DHEA-sulfate becomes available, and based on the above findings, likely achieves an intracellular peak in the mid-luteal subphase, the kidney and the colon—two of the non-endocrine human tissues which favor sulfation [39,40,41]—expedite excretion of DHEA-sulfate, which is the likely mechanism of our observed effect. If true, this effect, in fact, would occur in parallel with the well-known mid-luteal increase in renal blood flow as well as glomerular filtration rate [42].

The present study is impactful because it is the first characterization of DHEA, DHEA-sulfate, and the ratio between the two steroid hormones across the entire menstrual cycle, bringing important considerations to women’s health research. Previous studies comparing menstrual cycle phase (follicular versus luteal) found no difference [43,44], or higher levels in the follicular phase [45]. By deep mapping DHEAS trajectories across the menstrual cycle, we show that the trajectory of DHEA-sulfate in the luteal phase is inverse to that of estradiol and progesterone, with the highest contrast at the time of peak estradiol and progesterone in the mid-luteal subphase. This has relevance in the design and interpretation of clinical research evaluating associations between DHEA-sulfate levels and various disease states and substance intake, such as alcohol consumption [46]; breast [47,48] and skin [49] cancer risk; smoking; and cardiovascular risk [50,51], including atherosclerosis [52]. When conducted in reproductive-age women, the results of our analysis show that these studies should control for menstrual cycle effects. Future studies should assess levels of DHEA-sulfate across the menstrual cycle in women with reproductive disorders, as well as disorders with known associations with DHEA-sulfate.

Our study is limited by its sample size, which, due to the current cost of mass spectrometry steroid hormone analytic methodology, had to be kept at a minimum. Therefore, the study’s results must be interpreted with caution. However, a study sample size must be weighed against sources of bias; specifically, whether bias is introduced from a sample of participants of mixed biological background, which may skew the study results. Indeed, our study population, among other study criteria, was free of prescription and illicit drug intake, did not smoke or consume large amounts of alcohol, and did not have a current DSM 5-defined anxiety or depression. The strengths of our study are the implementation of the mass spectrometry methodology, which is the gold standard for steroid hormone measurement [53], and classification of study participants to the same menstrual cycle window by implementing a complex, validated study protocol to realign and impute the study data. Hence, the present study generated a comprehensive dataset of high quality.

In conclusion, the levels of DHEA-sulfate are the lowest in the mid-luteal subphase, while the levels of DHEA do not vary across the menstrual cycle. The present finding maps enzymatic activities in reproductive-age women and advances our understanding of circulating steroid hormone levels of critical importance to human health.

## Figures and Tables

**Table 1:** DHEA-Sulfate timepoint pairwise comparisons.

Timepoint Comparison		Class	Difference	*p* Value	Adjusted *p* Value	Significance
Early Follicular	Mid-Follicular	Realigned	−1.5863	0.1340	0.2010	ns
Early Follicular	Periovulatory	Realigned	−1.0568	0.3090	0.3635	ns
Early Follicular	Early Luteal	Realigned	3.8236	0.0020	0.0050	**
Early Follicular	Mid-Luteal	Realigned	5.6265	0.0001	0.0004	***
Early Follicular	Late Luteal	Realigned	0.6995	0.5000	0.5357	ns
Mid-Follicular	Periovulatory	Realigned	0.6328	0.5360	0.5360	ns
Mid-Follicular	Early Luteal	Realigned	5.0998	0.0002	0.0008	**
Mid-Follicular	Mid-Luteal	Realigned	7.1353	0.0000	0.0001	***
Mid-Follicular	Late Luteal	Realigned	1.7301	0.1090	0.1817	ns
Periovulatory	Early Luteal	Realigned	3.3780	0.0050	0.0107	**
Periovulatory	Mid-Luteal	Realigned	5.7557	0.0001	0.0004	***
Periovulatory	Late Luteal	Realigned	1.4247	0.1850	0.2523	ns
Early Luteal	Mid-Luteal	Realigned	2.1868	0.0510	0.0956	ns
Early Luteal	Late Luteal	Realigned	−1.0646	0.3150	0.3635	ns
Mid-Luteal	Late Luteal	Realigned	−4.9848	0.0006	0.0017	**
Early Follicular	Mid-Follicular	Imputed	−1.5191	0.1528	0.2495	ns
Early Follicular	Periovulatory	Imputed	0.0491	0.9619	0.9619	ns
Early Follicular	Early Luteal	Imputed	2.2421	0.0586	0.1256	ns
Early Follicular	Mid-Luteal	Imputed	4.5609	0.0004	0.0026	**
Early Follicular	Late Luteal	Imputed	0.8492	0.4148	0.4786	ns
Mid-Follicular	Periovulatory	Imputed	1.0254	0.3411	0.4651	ns
Mid-Follicular	Early Luteal	Imputed	2.9968	0.0312	0.0937	ns
Mid-Follicular	Mid-Luteal	Imputed	5.4979	0.0000	0.0007	**
Mid-Follicular	Late Luteal	Imputed	1.8137	0.0910	0.1706	ns
Periovulatory	Early Luteal	Imputed	2.3709	0.0496	0.1240	ns
Periovulatory	Mid-Luteal	Imputed	3.7107	0.0045	0.0168	*
Periovulatory	Late Luteal	Imputed	0.7108	0.5007	0.5365	ns
Early Luteal	Mid-Luteal	Imputed	1.5299	0.1664	0.2495	ns
Early Luteal	Late Luteal	Imputed	−0.8781	0.4139	0.4786	ns
Mid-Luteal	Late Luteal	Imputed	−4.7800	0.0014	0.0068	**

* *p* ≤ 0.05 ** *p* ≤ 0.01 *** *p* ≤ 0.001 ns=not significant.

## Data Availability

The data presented in this study are available on request from the corresponding author. The data are not publicly available due to preserve scientific integrity of research methodology.

## Supplementary Materials

The following supporting information can be downloaded at: https://www.mdpi.com/article/10.3390/metabo12100941/s1, Figure S1: Circulating level changes for dehydroepiandrosterone in the original, realigned, and imputed datasets; Figure S2: Circulating level changes for the ratio of dehydroepiandrosterone-sulfate: dehydroepiandrosterone in the original, realigned, and imputed datasets; Table S1: Algorithm to schedule clinic visits (in days) based on average self-reported cycle length in the BioCycle Study (Mumford et al., 2011); Table S2: Algorithm for aligning the day of the luteinizing hormone (LH) surge (visit 4) on the standardized LH surge visit (O2) (based on the Biocycle study, Mumford et al., 2011); Table S3: DHEA timepoint pairwise comparisons; Table S4: DHEAS:DHEA ratio timepoint pairwise comparisons.
