# Supplementary material for: Reduced Dehydroepiandrosterone-Sulfate Levels in the Mid-Luteal Subphase of the Menstrual Cycle: Implications to Women’s Health Research
Source: Metabolites. 2022 Oct 4;12(10):941. doi: 10.3390/metabo12100941 (PMC9611561; doi:10.3390/metabo12100941)
Supplement: Supplementary file 1 [file metabolites-12-00941-s001.zip › metabolites-1943585-supplementary.pdf]

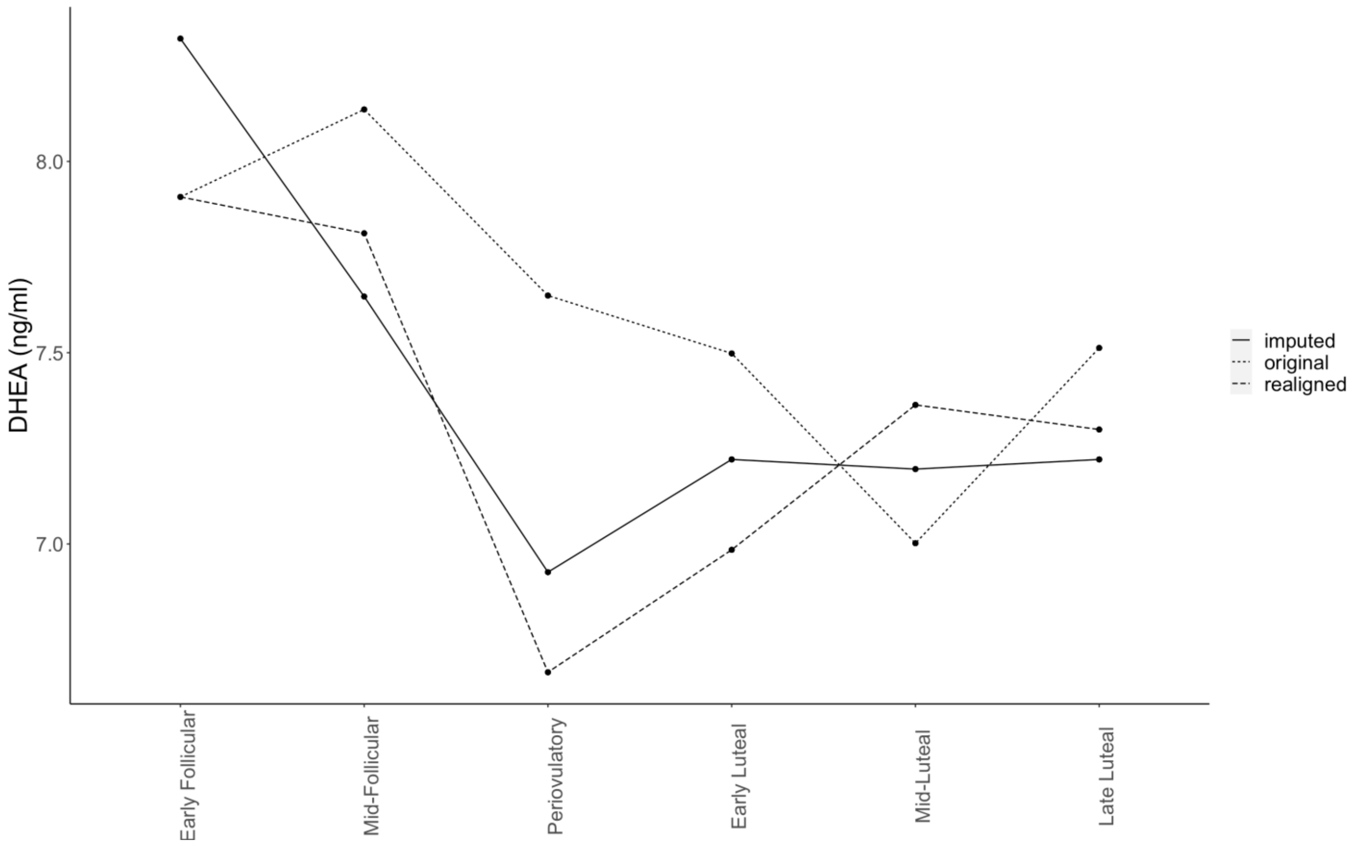

Figure S1. Circulating level changes for dehydroepiandrosterone in the original, realigned, and imputed datasets.

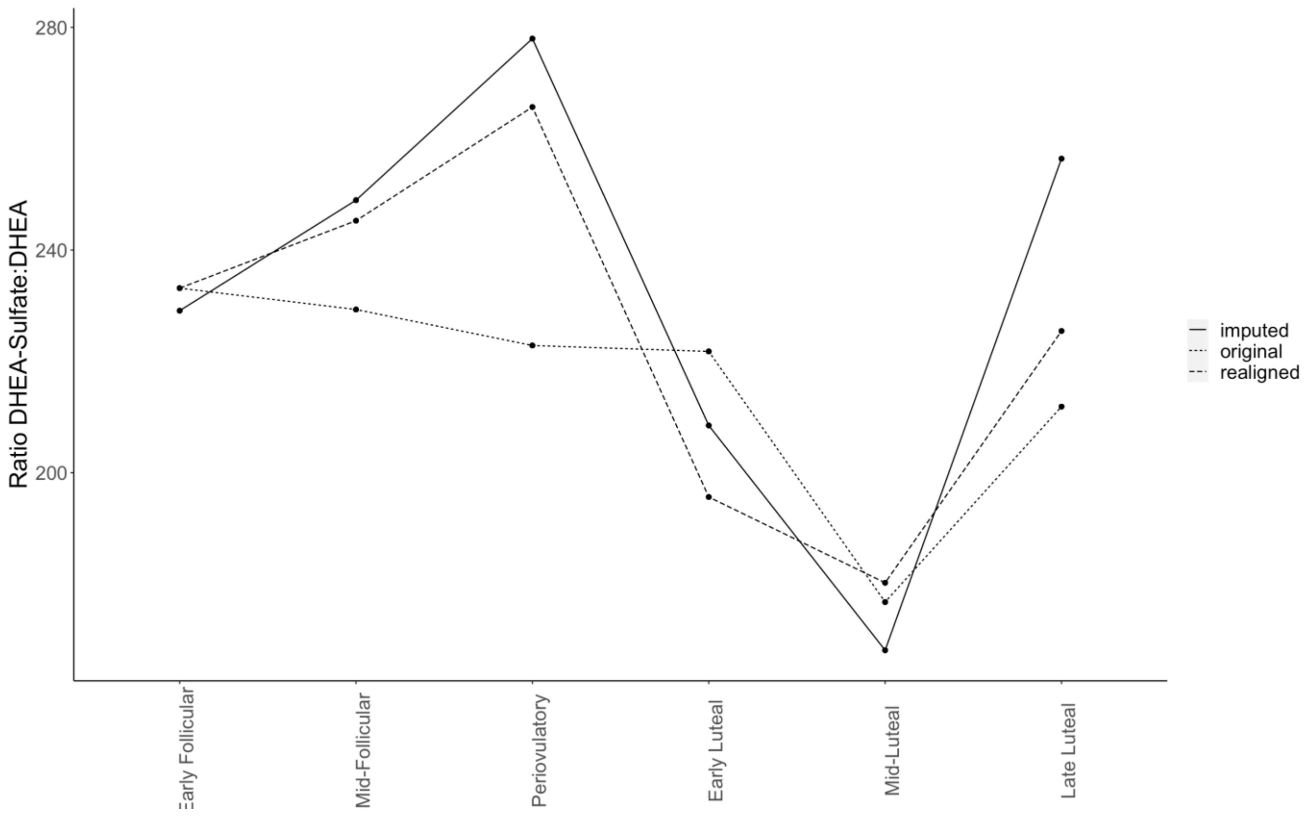

Figure S2. Circulating level changes for the ratio of dehydroepiandrosterone-sulfate: dehydroepiandrosterone in the original, realigned, and imputed datasets.

Table S1. Algorithm to schedule clinic visits (in days) based on average self-reported cycle length in the BioCycle Study (Mumford et al., 2011).

Average cycle length (days)	Visit 1 Menses (M)	Visit 2 Mid follicular (MF)	Visit 3 Periovulatory 1 (O1)	Visit 4 Periovulatory 2[a] (O2)	Visit 5 Periovulatory 3 (O3)	Visit 6 Early luteal (L1)	Visit 7 Mid luteal (L2)	Visit 8 Late luteal (L3)
21	2	4	5	6	7	11	15	20
22	2	4	6	7	8	12	16	21
23	2	5	7	8	9	13	17	22
24	2	5	8	9	10	14	18	23
25	2	6	9	10	11	15	19	24
26	2	6	10	11	12	16	20	25
27	2	7	11	12	13	17	21	26
28	2	7	12	13	14	18	22	27
29	2	7	13	14	15	19	23	28
30	2	8	14	15	16	20	24	29
31	2	8	15	16	17	21	25	30
32	2	9	16	17	18	22	26	31
33	2	9	17	18	19	23	27	32
34	2	10	18	19	20	24	28	33
35	2	10	19	20	21	25	29	34

[a]Luteinising hormone surge.

Table S2. Algorithm for aligning the day of the luteinising hormone (LH) surge (visit 4) on the standardised LH surge visit (O2) (based on the Biocycle study, Mumford et al., 2011).

		**Standardised cycle phase[b]**								
**LH Detection**	**LH peak occurred on visit (*n*)**	**Menses (M)**	**Mid follicular (F1)**	**Periovulatory 1 (O1)**	**Periovulatory 2 (LH surge; O2)**	**Periovulatory 3 (O3)**	**Early luteal (L1)**	**Mid luteal (L2)**	**Late luteal (L3)**
Early LH peak	Visit 3 (115) *Periovulatory 1*	Visit 1	Visit 2	*Missing[a]*	Visit 3	*Average* of visits 4 & 5	Visit 6	Visit 7	Visit 8
Correctly timed	Visit 4 (139) *Periovulatory 2 (LH surge)*	Visit 1	Visit 2	Visit 3	Visit 4	Visit 5	Visit 6	Visit 7	Visit 8
Late LH peak	Visit 5 (98) *Periovulatory 3*	Visit 1	Visit 2	*Average* of visits 3 & 4	Visit 5	*Missing*	Visit 6	Visit 7	Visit 8
	Visit 6 (30) *Early luteal phase*	Visit 1	*Average* of visits 3, 4 & 5	*Missing*	Visit 6	*Missing*	Visit 7	Visit 8	*Missing*
	Visit 7 (19) *Mid luteal phase*	Visit 1	*Average* of visits 3, 4 & 5	*Missing*	Visit 7	*Missing*	Visit 8	*Missing*	*Missing*
	Visit 8 (5) *Late luteal phase*	Visit 1	*Average* of visits 3, 4 & 5	*Missing*	Visit 8	*Missing*	*Missing*	*Missing*	*Missing*

[a] Missing' indicates that after realignment there was no serum collection (visit) during that phase of the cycle such that the reclassified visit was set to missing.
[b] If we assume a standard 28-day cycle, the standardised cycle phases would correspond to approximately days 2, 7, 12, 13, 14, 18, 22 and 27, respectively. Alternatively, these visits could be referenced from the day of the LH surge of a 28-day cycle as: onset of menses

until day -8 relative to the LH surge, day -7 to -2, day -1, day 0, day +1, day +2 to day +7, day +8 to day +11, day +12 to end of cycle.

Table S3. DHEA timepoint pairwise comparisons.

Timepoint Comparison		Class	Difference	*p* value	Adjusted *p* value	Significance
Early Follicular	Mid-Follicular	Realigned	−0.1118	0.9120	0.9620	ns
Early Follicular	Periovulatory	Realigned	0.7262	0.4790	0.9518	ns
Early Follicular	Early Luteal	Realigned	0.9892	0.3390	0.9518	ns
Early Follicular	Mid-Luteal	Realigned	0.9402	0.3620	0.9518	ns
Early Follicular	Late Luteal	Realigned	1.2647	0.2320	0.9518	ns
Mid-Follicular	Periovulatory	Realigned	1.1652	0.2620	0.9518	ns
Mid-Follicular	Early Luteal	Realigned	1.3103	0.2130	0.9518	ns
Mid-Follicular	Mid-Luteal	Realigned	0.6815	0.5050	0.9518	ns
Mid-Follicular	Late Luteal	Realigned	0.6112	0.5520	0.9518	ns
Periovulatory	Early Luteal	Realigned	0.3972	0.6980	0.9518	ns
Periovulatory	Mid-Luteal	Realigned	−0.1119	0.9120	0.9620	ns
Periovulatory	Late Luteal	Realigned	−0.2392	0.8160	0.9620	ns
Early Luteal	Mid-Luteal	Realigned	−0.4938	0.6300	0.9518	ns
Early Luteal	Late Luteal	Realigned	0.4074	0.6930	0.9518	ns
Mid-Luteal	Late Luteal	Realigned	−0.0485	0.9620	0.9620	ns
Early Follicular	Mid-Follicular	Imputed	−0.0233	0.8135	0.9387	ns
Early Follicular	Periovulatory	Imputed	0.0740	0.4120	0.9387	ns
Early Follicular	Early Luteal	Imputed	0.0340	0.7378	0.9387	ns
Early Follicular	Mid-Luteal	Imputed	0.0732	0.5521	0.9387	ns
Early Follicular	Late Luteal	Imputed	0.0389	0.7152	0.9387	ns
Mid-Follicular	Periovulatory	Imputed	0.0973	0.3135	0.9387	ns
Mid-Follicular	Early Luteal	Imputed	0.0573	0.5681	0.9387	ns
Mid-Follicular	Mid-Luteal	Imputed	0.0965	0.3816	0.9387	ns
Mid-Follicular	Late Luteal	Imputed	0.0622	0.5535	0.9387	ns
Periovulatory	Early Luteal	Imputed	−0.0400	0.6871	0.9387	ns
Periovulatory	Mid-Luteal	Imputed	−0.0009	0.9947	0.9947	ns
Periovulatory	Late Luteal	Imputed	−0.0351	0.7715	0.9387	ns
Early Luteal	Mid-Luteal	Imputed	0.0392	0.7453	0.9387	ns
Early Luteal	Late Luteal	Imputed	0.0049	0.9599	0.9947	ns
Mid-Luteal	Late Luteal	Imputed	−0.0343	0.7628	0.9387	ns

ns= not significant.

Table S4. DHEAS:DHEA ratio timepoint pairwise comparisons.

Timepoint Comparison		Class	Difference	*p* value	Adjusted *p* value	Significance
Early Follicular	Mid-Follicular	Realigned	0.7560	0.4610	0.5319	ns
Early Follicular	Periovulatory	Realigned	0.7638	0.4580	0.5319	ns
Early Follicular	Early Luteal	Realigned	−1.4760	0.1640	0.3075	ns
Early Follicular	Mid-Luteal	Realigned	−1.8663	0.0850	0.3043	ns
Early Follicular	Late Luteal	Realigned	0.0239	0.9810	0.9810	ns
Mid-Follicular	Periovulatory	Realigned	0.5915	0.5630	0.6032	ns
Mid-Follicular	Early Luteal	Realigned	−1.5623	0.1420	0.3043	ns
Mid-Follicular	Mid-Luteal	Realigned	−4.0041	0.0010	0.0150	*
Mid-Follicular	Late Luteal	Realigned	−1.0304	0.3230	0.4636	ns
Periovulatory	Early Luteal	Realigned	−1.5690	0.1410	0.3043	ns
Periovulatory	Mid-Luteal	Realigned	−2.9404	0.0120	0.0900	ns
Periovulatory	Late Luteal	Realigned	−1.1812	0.2650	0.4417	ns
Early Luteal	Mid-Luteal	Realigned	−1.6479	0.1280	0.3043	ns
Early Luteal	Late Luteal	Realigned	1.0080	0.3400	0.4636	ns
Mid-Luteal	Late Luteal	Realigned	1.7275	0.1150	0.3043	ns
Early Follicular	Mid-Follicular	Imputed	0.0833	0.5404	0.6235	ns
Early Follicular	Periovulatory	Imputed	0.1215	0.3998	0.6197	ns
Early Follicular	Early Luteal	Imputed	−0.1290	0.4284	0.6197	ns
Early Follicular	Mid-Luteal	Imputed	−0.3074	0.0374	0.1872	ns
Early Follicular	Late Luteal	Imputed	−0.0269	0.8090	0.8090	ns
Mid-Follicular	Periovulatory	Imputed	0.0382	0.7622	0.8090	ns
Mid-Follicular	Early Luteal	Imputed	−0.2123	0.1113	0.2988	ns
Mid-Follicular	Mid-Luteal	Imputed	−0.3907	0.0114	0.1717	ns
Mid-Follicular	Late Luteal	Imputed	−0.1102	0.4580	0.6197	ns
Periovulatory	Early Luteal	Imputed	−0.2504	0.1195	0.2988	ns
Periovulatory	Mid-Luteal	Imputed	−0.4289	0.0278	0.1872	ns
Periovulatory	Late Luteal	Imputed	−0.1484	0.3565	0.6197	ns
Early Luteal	Mid-Luteal	Imputed	−0.1785	0.3315	0.6197	ns
Early Luteal	Late Luteal	Imputed	0.1021	0.4957	0.6197	ns
Mid-Luteal	Late Luteal	Imputed	0.2805	0.0791	0.2967	ns

ns= not significant.
